# Anxiety and Depression Levels and Coping Strategies among Polish Healthcare Workers during the COVID-19 Pandemic

**DOI:** 10.3390/ijerph20043319

**Published:** 2023-02-14

**Authors:** Natalia Budzyńska, Joanna Moryś

**Affiliations:** Department of Clinical Psychology, Faculty of Health Sciences, Medical University of Gdańsk, 80-210 Gdańsk, Poland

**Keywords:** COVID-19, SARS-CoV-2, healthcare workers, anxiety, depression symptoms, coping strategies

## Abstract

During the COVID-19 pandemic, medical staff were commonly exposed to stress, work under pressure, and long shifts, and may have experienced a fear of infecting loved ones or a fear for their own health. All of these factors may have increased the likelihood that healthcare workers will experience the symptoms of depression, anxiety, or other mental health disorders. In this cross-sectional study, a group of respondents was gathered from the employees of 78 hospitals in Poland. A questionnaire was completed electronically by 282 people, aged between 20 and 78 years. The study used the Hospital Anxiety and Depression Scale (HADS) and the MiniCOPE questionnaire to examine anxiety and depression symptoms, and coping strategies, respectively. With age, the respondents declared fewer symptoms of anxiety and tended to have milder symptoms of depression. Participants with chronic illnesses, mood disorders, or anxiety disorders also reported higher levels of anxiety and depression symptoms. More than 20% of healthcare workers felt the need to consult with a psychologist. In the entire group of healthcare professionals surveyed, the most commonly used strategies for coping with stress were “denial”, “psychoactive drug and alcohol use”, and “cessation of activities”, while the least used strategy was “acceptance”. Given the most commonly used strategies in the surveyed group of healthcare professionals, they may be predictors for a deterioration in mental state in the long run. The obtained results also suggest that it is likely that pre-existing health problems had a greater impact on the mental health of medical staff during the COVID-19 pandemic than the profession itself. Therefore, taking care of the well-being and mental health of healthcare workers should be a priority for employers.

## 1. Introduction

At the beginning of the twenty-first century, four major pandemics broke out worldwide. In 2003 [1], the first pandemic caused by a virus from the coronavirus family occurred. This pandemic affected 29 countries around the world [2,3]. In 2009, the first influenza A (H1N1) epidemic broke out in Mexico, affecting 208 countries [4]. In 2012, a second coronavirus pandemic broke out in the Arabian Peninsula—MERS [5,6,7]. In December 2019, another pandemic, and the third one caused by a human coronavirus, broke out in Wuhan, China [5]. 

Despite these experiences, the world was not prepared for what the emergence of the SARS-CoV-2 virus brought with it [8]. Certainly, the health care system in Poland was not prepared for this and struggled significantly with staff shortages [9,10], equipment shortages (especially shortages of personal protective equipment) [9,11], and underfunding. In Poland, hospital wards closed, and scheduled admissions, procedures, surgical operations, and treatments were suspended. Only life-saving procedures took place. Specialist wards were also closed and transformed into COVID-19 wards. In the subsequent stages of the pandemic’s development, special hospitals were created to treat only patients with COVID-19. These were created in exhibition halls and, in Warsaw, even in the National Stadium.

All over the world, medical workers, including doctors, nurses, paramedics, and other personnel, stood on the front line of the fight against the COVID-19 pandemic [12]. As a result, they have become one of the groups most at risk of developing mental health complications [13]. Long shifts, exposure to death [14], working in harsh conditions, stress [15], exhaustion [16], fear of contact with an infected person [17], concerns about personal health, fear of infecting loved ones [11,14,16], and dealing with lockdown challenges (e.g., household responsibilities and worries, childcare, remote learning) are only some of the challenges experienced by health care workers during the COVID-19 pandemic [11,14]. In addition, frequent staff or equipment shortages exerted added pressure [14]. Medical personnel exposed to stress and working under pressure are vulnerable and at risk of experiencing the symptoms of depression, anxiety, or other mental health problems [15,18].

Many healthcare workers in Poland have also experienced stigmatization, social exclusion, or even attacks, such as name-calling, broken car windows, or punctured tires. Individuals working in wards or hospitals dedicated to the treatment of COVID-19 patients were particularly exposed to such attacks [10,11]. These factors have created a very difficult situation for healthcare workers during the pandemic in Poland and worldwide. To date, research conducted in Poland clearly shows that healthcare workers have experienced increased symptoms of anxiety and depression [11,18], are more likely to suffer from sleep disorders [13], and over 72% believe that their mental health is impaired [10]. A significant increase in alcohol consumption among Polish healthcare workers has also been reported [12]. As with other groups in the population, among healthcare workers, the groups at greatest risk for the development of mental health issues are women, younger and older individuals, people with less professional experience, and those in direct contact with infected patients [12].

The aim of the current study was to assess the level of anxiety and depression symptoms in healthcare workers who have worked under the exceptional conditions created by the COVID-19 pandemic for over 1.5 years. In addition, this study aimed to examine the strategies used by healthcare workers to cope with the stress experienced under these conditions. We also set out to explore how healthcare professionals with previous mental health problems are dealing with the current situation.

## 2. Materials and Methods

### 2.1. Participants and the Research Plan

This cross-sectional study was conducted among healthcare professionals working in hospitals across Poland. A group of respondents was gathered formally. In the period between August 2021 and March 2022, invitations for employees to complete an electronic, anonymous questionnaire survey were sent to the management of 350 hospitals throughout Poland. We received 108 responses to the invitations, of which 78 institutions agreed to participate in the study. The link to the questionnaire was sent via the internal IT systems of the institutions or by e-mail, along with an internal newsletter containing information about the study and a request to complete the survey.

A full cross-section of hospital employees was invited to participate in the study. This included not only people working directly with patients, such as doctors, nurses, physiotherapists, or other medical staff, but also other personnel, such as laboratory diagnosticians and administrative employees. The inclusion criteria were a minimum age of 18 years and currently working in a hospital. As the questionnaire required answers to all questions (otherwise they were not recorded in the database), only individuals who completed the entire questionnaire were included in the study.

### 2.2. Methods Used

This study used the Hospital Anxiety and Depression Scale (HADS), the MiniCOPE scale, and a demographic questionnaire.

A version of the HADS by Zigmond and Snaith [19] was developed in Polish by the team of M. Majkowicz, K. De Walden-Gałuszko, and G. Chojnacka-Szawłowska. It is a screening method used to identify the symptoms of anxiety and depression, consisting of 7 items examining the symptoms of depression and 7 relating to anxiety. For both scales, scores between 0 and 7 points indicate an absence of disturbances, between 8 and 10 points indicate limited states, and between 11 and 21 points indicate the presence of disorders [20,21].

In validation studies of the original version of the scale, the Spearman’s rank correlation coefficients between test positions and the overall scores on a given scale were statistically significant (at least *p* < 0.01) and ranged from +0.76 to +0.41 for the anxiety scale. The correlation coefficients for the depression scale ranged between +0.60 and +0.30 (*p* < 0.02) [21]. In studies of translations of the questionnaire, Cronbach’s alpha for the anxiety scale ranged from 0.68 to 0.93 (mean 0.83), and for the depression scale, from 0.67 to 0.90 (mean 0.82). Internal consistency in different translations confirms the reliability of the HADS scale [22].

The MiniCOPE scale is a tool used to measure coping strategies in various situations. This questionnaire consists of 28 items capturing 14 scales. Each item is scored from 0 (I almost never do this) to 3 (I almost always do so), and assesses the frequency of occurrence of a given behavior in difficult situations. In the original version, Cronbach’s alpha ranged from 0.48 to 0.94. When remeasured after an interval of 6 weeks, the constancy index ranged from 0.45 to 0.82. The half-reliability of the Polish version of the MiniCOPE inventory was calculated, obtaining a Guttman index of 0.87. Internal consistency for most of the scales is about 0.70, and similar indicators were obtained for the Polish version. The factor loadings for most of the items were considered satisfactory [23].

The respondents were asked questions about their gender, age, place of residence (village, city of over 10,000, city of 10,000–50,000, city of 50,000–100,000, city of 100,000–200,000, city of over 200,000), education (secondary education, bachelor’s degree, engineering, master’s degree, doctorate), occupation (administrative staff, nurse, doctor, other medical personnel, other medical specialists, laboratory diagnostician, midwife, physiotherapist, medical technician), physical (are you chronically ill? Yes/no) and mental health (have you ever been diagnosed with depression or any other mood disorder? Yes/no; have you ever been diagnosed with an anxiety disorder? Yes/no; have you ever been diagnosed with other mental disorders/diseases? Yes/no), history of SARS-CoV-2 infection (have you received a positive result for a SARS-CoV-2 infection? Yes/no), as well as hospitalization in connection with the infection (have you been hospitalized due to COVID-19 disease? Yes/no/not applicable) and work in a ward dedicated to patients with COVID-19 (yes/yes, but I do not work in one anymore/no/not applicable). A question was also asked about the need to consult a psychologist in connection with the work performed (do you feel a need to consult with a psychologist in connection with your work? Definitely yes/likely yes/I don’t know/likely no/definitely no).

### 2.3. Statistical Analysis

To analyze the relationships between age, education, place of residence, and the levels of symptoms of anxiety and depression, Pearson’s correlation coefficients and non-parametric Kruskal–Wallis ANOVAs were used. In addition, the results were verified by a model approach (a system of variables together) using regression analysis. In order to analyze the relationships between individual variables, the results were correlated. For this analysis, Pearson’s correlation coefficients were also used. The results were also analyzed for group differences. Groups were compared using the *t*-test for independent groups. Due to a disproportion in the number of compared groups, the results were also verified using the Mann–Whitney *U* test.

The distributions for each variable were tested for normality using the Kolmogorov–Smirnov test. If the test indicated a non-normal distribution, the results obtained were also verified with appropriate non-parametric tests (e.g., Kruskal–Wallis test, Mann–Whitney *U* test).

The values for continuous variables were calculated according to the keys for the measurement tools (most often as the sum of points assigned to the appropriate scale in the test) or read from the declarations of the participants (e.g., age). In the tables, the data are expressed as mean value, standard deviation, median, dominant, and maximum and minimum score. If there was a need for a general description of all results for the entire group of participants, the raw data was converted into a scale for the appropriate test (most often these were stens). Categorical variables were counted according to a given criterion. When there was such a need, a criterion consistent with the research questions posed was used.

For statistical analyses, SPSS (IBM, Armonk, NY, USA) versions 25 and 26 were used.

## 3. Results

During the study period, the survey was completed by 282 people aged between 20 and 78 years. The average age of the respondents was 43 years, with a median age of 44 years. 86.5% (n = 243) of the respondents were women and 13.5% (n = 38) were men. The vast majority of the respondents were individuals who had higher education (84.26%), and the remaining individuals received a secondary education (15.74%). In the study group of medical workers, 34.2% of the participants had been infected with the SARS-CoV-2 virus; of these, only 2 were hospitalized for the infection. In the group of respondents, 23.5% of participants had worked in a ward dedicated to patients with COVID-19.

### 3.1. Levels of Anxiety and Depression Symptoms (HADS Scores)

**Figure 1:**
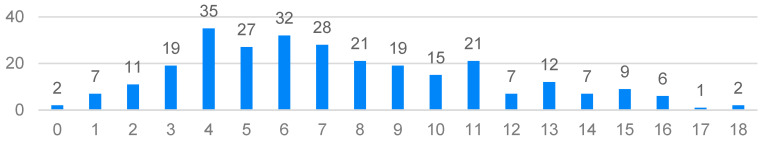
Distribution of the Hospital Anxiety and Depression Scale (HADS) anxiety scores.

**Figure 2:**
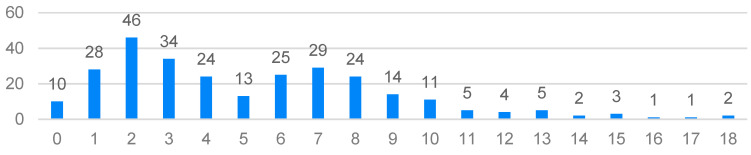
Distribution of the HADS depression scores.

On the HADS scale, the results were in the range from 0 to 18 points (the maximum score on this scale is 21 points). In terms of the symptoms of anxiety and depression in the study group, the average results did not particularly differ from the norm. The mean value for anxiety symptoms was 7.44 (SD = 3.897) and nearly 65% of the respondents obtained a result within 0–8 points. For the symptoms of depression, the mean score was 5.25 (SD = 3.719) and nearly 83% of the participants scored in the range of 0–8. On the anxiety scale, the most common score was 4 points (n = 35), while on the depression scale, the most common score was 2 points (n = 46). Apart from the borderline results, 16% of the respondents had scores indicating a high degree of anxiety symptoms (12–18 points) and 6.4% reported elevated symptoms of depression (12–18 points). However, it is worth bearing in mind the noticeable variations in the results among the participants, and that there were clearly more severe symptoms of anxiety compared to the symptoms of depression (Figure 1 and Figure 2).

In order to answer the question “What healthcare workers had the highest levels of anxiety and depression symptoms during the pandemic, excluding participants with health problems?”, an analysis of the HADS scores (n = 172) was carried out that excluded people with chronic diseases and individual representatives of particular professions (n = 1; paramedic, student).

The collected data (Table 1) indicate that the highest levels of anxiety were reported by laboratory diagnosticians, midwives, and other medical personnel, and the lowest levels by physiotherapists and medical technicians. It is worth noting, however, that the results do not indicate very strong feelings (mean scores above eight are the so-called limit values indicating moderate symptoms of anxiety). In the case of depression, all of the results are within the normal range, with the highest scores reported by midwives and the lowest by other medical specialists.

### 3.2. The Relationships between Age, Education, Place of Residence, and the Level of Anxiety and Depression Symptoms

A statistically significant, negative relationship between age and the level of anxiety (on the verge of significance—the level of depression) was obtained. With age, the participants reported fewer anxiety symptoms and tended to have milder symptoms of depression (Table 2).

No statistically significant differences in terms of education or place of residence were found (Kruskal–Wallis).

### 3.3. Health Problems and the Level of Anxiety and Depression Symptoms

Six statistically significant (positive) relationships between the variables were observed. Chronically ill participants and those with mood disorders or anxiety disorders reported higher levels of anxiety and depression symptoms during the COVID-19 pandemic (Table 3).

### 3.4. Comparisons of Anxiety and Depression Symptoms in Participants with and without Chronic Diseases

There were statistically significant differences in the levels of anxiety and depression symptoms between the compared groups. Participants declaring previously diagnosed chronic diseases indicated significantly more symptoms of anxiety and depression during the COVID-19 pandemic (Table 4).

### 3.5. Comparisons of Anxiety and Depression Symptoms in Participants with and without Depression or Mood Disorders

There were statistically significant differences in the levels of anxiety and depression symptoms between the compared groups. Participants declaring diagnosed depression or mood disorders indicated significantly more symptoms of anxiety and depression during the COVID-19 pandemic (Table 5).

### 3.6. Comparisons of Anxiety and Depression Symptoms in Participants with and without Anxiety Disorders

Statistically significant differences in the levels of anxiety and depression symptoms were obtained between the compared groups. Healthcare workers reporting diagnosed anxiety disorders indicated significantly more symptoms of anxiety and depression during the COVID-19 pandemic (Table 6).

### 3.7. Comparisons of Anxiety and Depression Symptoms in Participants with and without Mental Illness

No statistically significant differences were observed. Due to the large disproportion in the number of compared groups, the result was also verified using the Mann–Whitney *U* test.

#### 3.7.1. Consultations with a Psychologist

**Figure 3:**
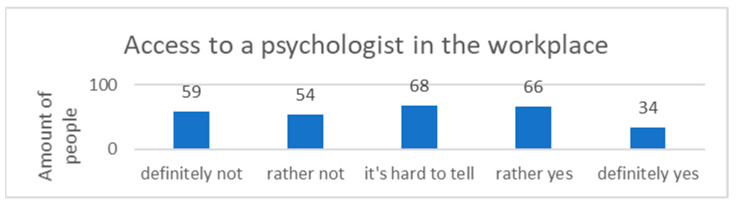
Access to a psychologist in the workplace.

Lack of access to a psychologist in the workplace was reported by 40.2% (n = 113) of the participants. In total, 24.2% (n = 68) of the respondents did not know whether consultations with a psychologist were available for employees, while 35.6% (n = 100) declared that there was a possibility of a consultation with a psychologist in the workplace (Figure 3).

**Figure 4:**
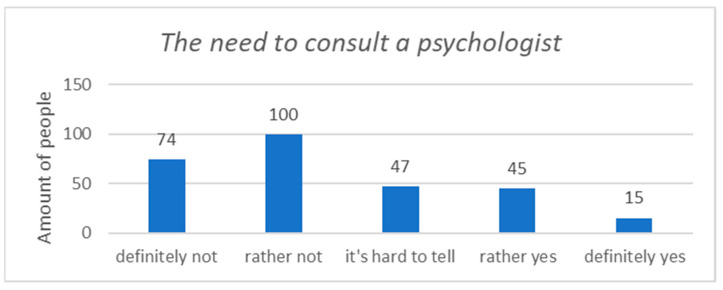
The need to consult a psychologist in connection with the work performed.

Most of the respondents (61.9%, n = 174) did not feel the need to consult a psychologist in connection with their work, while 21.3% (n = 60) of healthcare workers felt such a need (Figure 4).

Statistically significant (positive) relationships were observed. Respondents who felt the need to consult with a psychologist reported an increased level of anxiety and depression symptoms (Table 7).

Six statistically significant (positive) relationships were obtained between the analyzed variables. Respondents reporting a strong need for contact with a psychologist showed a tendency to refrain from action, stop dealing with the problem, run away from it, distract their attention in stressful situations, focus on emotions, and increase use of alcohol or other drugs (Table 8).

#### 3.7.2. Dominant Coping Strategies (MiniCOPE Scores)

The highest mean scores were obtained for “denial”, “use of psychoactive drugs and alcohol”, and “cessation of activities”. The lowest score was obtained for “acceptance”. The strategies “turning to religion”, “denial”, “cessation of action”, and “escape into drugs” dominated (Table 9).

#### 3.7.3. The Relationships between Age and Coping Strategies

Statistically significant, positive correlations were found between the ages of the participants and the following strategies: seeking emotional support, refraining from action, focusing on emotions and their discharge, negation, distraction, cessation of activities, and sense of humor (Table 10). The strategy “a turn to religion” was negatively correlated with participant age.

#### 3.7.4. Associations between Anxiety and Depression Levels and Coping Strategies

In total, 15 statistically significant relationships (2 negative and 13 positive) were observed. Surveyed medical personnel reporting high levels of anxiety and depression symptoms showed a tendency to deny problems and refrain from actions or even stop them (especially for participants with increased symptoms of depression). In addition, participants with increased symptoms of anxiety did not want to accept their situation, and depressed individuals tended to distract their attention, escape from trouble and stress, use psychoactive substances, and show a reduced sense of humor (Table 11).

#### 3.7.5. Associations between the Presence of Chronic Health Problems and Coping Strategies

Eight statistically significant (positive) correlations were obtained. The examined chronically ill patients sought instrumental support, and participants reporting mood disorders or depression, as well as anxiety disorders, showed a tendency to stop actions and use alcohol or other substances. On the other hand, respondents reporting mental illnesses particularly emphasized the use of alcohol or other drugs (Table 12).

#### 3.7.6. Relationships between a Previous COVID-19 Diagnosis and Symptoms of Anxiety and Depression

The impact of a previous COVID-19 diagnosis in healthcare professionals on the symptoms of anxiety and depression was investigated.

A *t*-test was used for independent samples, with no statistically significant differences identified (Table 13).

#### 3.7.7. Experience Working in a COVID-19 Ward and Symptoms of Anxiety and Depression

An ANOVA was performed, and the results were verified using a Kruskal–Wallis test. No statistically significant differences were observed (Table 14).

## 4. Discussion

The COVID-19 pandemic has undoubtedly affected the mental health of entire communities [15], increasing the symptoms of anxiety and depression [15,18,24], stress, and inducing feelings of loneliness [15]. It also had an impact on healthcare workers; although, in the current study group, the symptoms of anxiety and depression were within the normal range. It was also found that the most frequently used strategies for coping with stress during the pandemic are those from the nonadaptive group.

After about a year and a half of the pandemic, both in terms of the symptoms of anxiety and depression in the studied group of Polish healthcare workers, average results were obtained that did not differ particularly from the norm. In terms of anxiety symptoms, nearly 65% of the respondents scored within the normal range. In the case of symptoms of depression, almost 83% scored within the normal range. In total, 23% of the respondents had scores indicating an increased severity of anxiety symptoms, and 8.15% had scores indicating an increased severity of depression symptoms. However, it is worth bearing in mind the notable diversity in the results, as clearly more severe symptoms of anxiety were observed compared to depression. Previous research has also indicated increased symptoms of anxiety and depression among healthcare workers during the COVID-19 pandemic [24,25]. The prevalence rate for anxiety symptoms reported in previous studies varied between 11.7% and 50% [24,26], while those observed for depression were between 13.7% and 42% [27,28,29]. The differences in rates across studies may have resulted from the characteristics of the course of infections in a given country, the conditions of the healthcare system, the precise moment of the pandemic when the study was carried out, and the measurement tools used. During the period in which this study was conducted, the daily number of SARS-CoV-2 infections was several hundred in August 2021, and gradually increased from several thousand in October, to about 28,000 daily infections in November. At the turn of 2021/2022, a slight decrease in infections was observed. However, at the end of January 2022, there was another increase in infections, reaching approximately 55,000 infections per day. Following this, the number of infections began to decrease gradually to several thousand infections per day in March 2022 [30].

With age, the respondents declared fewer symptoms of anxiety and showed a tendency toward milder symptoms of depression. This finding indicates that the most difficulties are experienced by young people. Other studies have also reported that a young age is one of the predictors of the appearance of the clinical signs of mental health disorders [26,28,31]. The sensitivity of this group is also emphasized by the observation that there was an increased psychiatric hospitalization rate for younger individuals during the pandemic [32,33,34]. In addition, younger people were also at an increased risk of committing suicide during this period [33,35,36]. This data further reinforces the findings that young people are particularly vulnerable to mental health disorders and suggests that they may need increased support during difficult times, such as during the pandemic and the associated social isolation.

Similar associations have also been reported in the general population [37,38]. It is difficult to find similar studies among healthcare professionals in pre-pandemic Poland. However, medical professions are perceived as stressful. The shift nature of the work, responsibility, work under pressure, staff shortages, and the needs and requirements of patients lead to medical professions being perceived as stressful, and the individuals in these professions are at risk of, among other things, depression and anxiety disorders [39,40]. The level of depressive symptoms among doctors in various studies is estimated at between 8.8% and 28.1% [41].

Participants with various health problems (both physical and mental) also declared significantly higher levels of anxiety and depression symptoms during the COVID-19 pandemic. Similar data have been obtained by other researchers [26,42,43]. In the context of coping strategies, chronically ill healthcare workers tended to seek instrumental support, while the participants declaring mood disorders/depression or anxiety disorders tended to stop activities and use alcohol or other psychoactive drugs. Respondents declaring mental illness particularly emphasized the use of stimulants.

The subjective need for a psychological consultation was also examined. The respondents who declared the need for a consultation with a psychologist in connection with their work during the COVID-19 pandemic also reported increased levels of anxiety and depression symptoms. Similar data was obtained by Benzakour [44] in a qualitative study analyzing the symptoms of medics who came forward for psychological or psychiatric consultations offered by the hospital where they worked. Psychological or psychiatric help was mainly sought by employees working in COVID-19 wards that were experiencing increased levels of anxiety and depression, with anxiety symptoms being a stronger predictor than those of depression [44]. In addition, in the context of the strategies used to cope with stress, the surveyed healthcare workers who felt the need to consult a psychologist showed a tendency to refrain from actions, stop dealing with the problem, run away from it, distract their attention in stressful situations, focus on emotions, and use alcohol or other psychoactive drugs.

The surveyed medical personnel that reported high levels of anxiety and depression symptoms showed a tendency to deny problems and refrain from actions or even stop them (especially individuals with increased symptoms of depression). In addition, participants with increased symptoms of anxiety did not want to accept their situation, and depressed participants tended to distract their attention, escape from trouble and stress, increase drug alcohol and drug use, and have a reduced sense of humor.

Polish research conducted at the initial stage of the pandemic also indicates that medics using nonadaptive strategies to cope with stress show more symptoms of mental health disorders [45].

In the entire group of healthcare professionals surveyed, the most commonly used strategies for coping with stress during the pandemic were “denial”, “psychoactive drug and alcohol use”, and “cessation of activities”, while the least used strategy was “acceptance”. However, contrary to the results obtained in our study, other studies by Polish researchers indicate that more medics used adaptive strategies of coping with stress [45]. For example, a study conducted in a group of Polish nurses showed that they prefer active strategies for coping with stress and focus on the problem, while they least often resort to psychoactive substances [46,47] or cease activities [47,48].

Correlations between anxiety or depression symptoms and certain coping strategies, such as denial, cessation of activities, or the use of psychoactive drugs and alcohol, indicated a high risk to the mental health of the participants who used these types of strategies in difficult situations. Considering that these were the most commonly used strategies in the surveyed group of healthcare workers, the results obtained may be a predictor of a deterioration in the mental state of healthcare workers in the long term.

Interesting results were obtained in the context of anxiety and depression symptoms in healthcare workers who had a previous COVID-19 diagnosis, and in those who worked in a COVID-19 ward. In both cases, no statistically significant differences were observed. Thus, for the group of healthcare workers surveyed, neither personal experience with COVID-19 or working in a COVID-19 ward had any impact on the levels of anxiety or depression symptoms. These results are surprising, as previous research has indicated that working in COVID-19 wards is a risk factor for poorer mental health and that medics who are more exposed to the consequences of the pandemic exhibit poorer mental health than other occupational groups [4,17,25,26,28,29,30,42,49,50]. These results seem to coincide with another study also conducted in Poland, in which the results suggested that it is likely that pre-existing health problems had a greater impact on mental health during the pandemic than the profession itself [51].

It also should be noted that the mental health of medics may reduce the efficiency of cognitive functions, lower the quality of their work, and impact their relationships with patients [39]. Therefore, it is extremely important to provide healthcare workers with access to psychological care. Psychological consultations at the workplace are beneficial for personal difficulties and can also provide support in the context of difficult conversations with patients or contact with a problem patient. There are many effective interventions that support mental health and build resistance to stress, including regular physical activity, relaxation techniques, mindfulness, or yoga. Taking care of the well-being and mental health of healthcare workers is a huge challenge, but it should be a priority for employers [52]. Several important factors to consider in the context of mental health for medical staff are overworking, too many patients per staff member, and too many working hours, all of which result from staff shortages. In connection with the above, it is, therefore, necessary to introduce systemic solutions to ensure the appropriate number of staff.

## 5. Conclusions

The current study showed that after a year and a half of the COVID-19 pandemic, anxiety and depression symptoms of the healthcare workers examined here were within the norm.

Among the study group, high levels of anxiety and depression symptoms were associated with the use of stress-coping strategies consisting of denying problems and refraining from actions or even stopping them (this was especially true for individuals with severe symptoms of depression). In addition, participants experiencing severe symptoms of anxiety showed difficulties with accepting their situation, while depressive individuals tended to distract, escape from trouble and stress, use stimulants, and possess a reduced sense of humor.

An interesting result is the fact that, in the study group, the mental health of healthcare workers in Poland is more influenced by previous health problems and not by working in the medical profession.

The stress coping strategies used by the surveyed medical staff indicate a high risk to mental health in the individuals who use such coping strategies in difficult situations. Considering that these were the most frequently used strategies in the surveyed group during the COVID-19 pandemic, the results obtained may be a predictor of a deterioration in the mental state of healthcare workers in the long run.

In addition, the obtained results indicate that the mental health of healthcare workers in Poland is influenced more by previous health problems, and not by practicing the medical profession.

### Limitations

It is also important to be aware of the limitations of the current study. One such limitation is the degree of heterogeneity in the group of respondents. For example, women were disproportionately overrepresented in the current sample (86.5%). While this may be an issue for the present research, in many studies women constitute a significant majority of the study group. In addition, several professional groups were very underrepresented in the current study, including midwives (n = 4) and physiotherapists (n = 3). The research methodology also did not make it possible to determine the number of individuals that the survey reached, or the number of people who started the questionnaire but did not complete it. As mentioned above, the study was conducted in 78 hospitals throughout Poland. However, fatigue, a lack of time, or oversaturation with the subject of the pandemic may have been responsible for the low number of responses received.

## Figures and Tables

**Table 1:** HADS scale results for selected occupational groups.

	Anxiety		Depression	
	M	SD	M	SD
Administrative staff (n = 56)	7.14	3.403	4.73	3.392
Nurse (n = 47)	6.70	4.032	4.98	3.931
Doctor (n = 23)	6.70	4.193	4.30	3.111
Other medical personnel (n = 16)	8.13	4.965	5.38	3.612
Other medical specialists (n = 14)	4.43	2.793	2.86	2.349
Laboratory diagnostician (n = 5)	8.20	2.168	6.40	4.278
Midwife (n = 4)	8.25	3.775	7.25	5.123
Physiotherapist (n = 3)	4.33	2.517	4.00	4.359
Medical technician (n = 2)	5.50	2.121	4.00	1.414

M—mean, SD—standard deviation.

**Table 2:** Correlations between the HADS scores and age.

	Age	Education	Place of Residence
Anxiety	−0.137 **	3.23	10.97
Depression	−0.101 *	1.74	10.63

** *p* < 0.05; * *p* < 0.1.

**Table 3:** Correlations between HADS scores and health problems.

	Anxiety	Depression
Chronic illness	0.122 **	0.119 **
Depression/mood disorders	0.150 **	0.167 ***
Anxiety disorders	0.215 ***	0.154 ***
Other mental illnesses	0.037	0.053

*** *p* < 0.01; ** *p* < 0.05.

**Table 4:** Comparisons of HADS scores for participants with and without chronic diseases.

	Diagnosed with Chronic Diseases		No Chronic Diseases				
	M	SD	M	SD	t	df	d
Anxiety	8.16	3.845	7.13	3.887	2.061 **	279	0.257
Depression	5.92	4.147	4.95	3.488	2.006 **	279	0.264

M—mean, SD—standard deviation, t—*t*-test value, df—degrees of freedom, d—Cohen’s effect size, ** *p* < 0.05.

**Table 5:** Comparisons of HADS scores for participants with and without depression or mood disorders.

	Depression or Mood Disorders		No Depression or Mood Disorders				
	M	SD	M	SD	t	df	d
Anxiety	8.78	3.624	7.19	3.902	2.535 **	279	0.438
Depression	6.67	3.873	4.97	3.634	2.832 ***	279	0.466

M –mean, SD—standard deviation, t—*t*-test value, df—degrees of freedom, d—Cohen’s effect size,*** *p* < 0.01; ** *p* < 0.05.

**Table 6:** Comparison of HADS scores for participants with and without anxiety disorders.

	Anxiety Disorders		No Anxiety Disorders				
	M	SD	M	SD	t	df	d
Anxiety	10.12	3.930	7.18	3.801	3.681 ***	279	0.758
Depression	7.08	3.673	5.07	3.681	2.611 ***	279	0.539

M—mean, SD—standard deviation, t—*t*-test value, df—degrees of freedom, d—Cohen’s effect size, *** *p* < 0.01.

**Table 7:** Correlations between HADS scores and the need for consultation with a psychologist.

	Anxiety	Depression
In connection with your work, do you feel the need to consult a psychologist?	0.458 ***	0.432 ***

*** *p* < 0.01.

**Table 8:** Correlations between the MiniCOPE scores and the need to consult a psychologist.

	In Connection with Your Work, Do You Feel the Need to Consult a Psychologist?
Active coping	0.095
Planning	0.041
Seeking instrumental support	0.115
Seeking emotional support	0.095
A turn to religion	0.025
Positive re-evaluation and development	−0.043
Refraining from action	0.233 ***
Acceptance	−0.106
Focusing on emotions and their discharge	0.170 ***
Denial	0.157 ***
Distraction	0.191 ***
Cessation of activities	0.328 ***
Use of alcohol or other means	0.197 ***
Sense of humor	0.013

*** *p* < 0.01.

**Table 9:** Results obtained from the MiniCOPE.

	M	SD	D	Min	Max
Active coping	3.92	1.827	4	0	6
Planning	2.84	1.539	2	0	6
Seeking instrumental support	3.47	1.454	4	0	6
Seeking emotional support	3.15	1.631	2	0	6
A turn to religion	3.88	2.087	6	0	6
Positive re-evaluation and development	2.80	1.557	2	0	6
Refraining from action	4.26	1.479	5	0	6
Acceptance	1.69	1.248	2	0	6
Focusing on emotions and their discharge	3.70	1.536	3	0	6
Denial	5.17	1.243	6	0	6
Distraction	3.59	1.381	4	0	6
Cessation of activities	4.61	1.311	6	0	6
Use of alcohol or other psychoactive substances	5.14	1.314	6	0	6
Sense of humor	4.16	1.173	5	0	6

M—mean, SD—standard deviation, D—dominant.

**Table 10:** Correlations between coping strategies and participant’s ages.

	Age
Active coping	−0.046
Planning	0.012
Seeking instrumental support	0.115
Seeking emotional support	0.166 *
A turn to religion	−0.163 *
Positive re-evaluation and development	−0.210
Refraining from action	0.176 *
Acceptance	−0.015
Focusing on emotions and their discharge	0.189 *
Denial	0.133 **
Distraction	0.141 **
Cessation of activities	0.167 *
Using alcohol or other psychoactive substances	0.073
Sense of humor	0.130 **

* *p* < 0.01; ** *p* < 0.05.

**Table 11:** Correlations between the HADS and MiniCOPE scales.

	Anxiety	Depression
Active coping	−0.09	−0.116
Planning	−0.035	0.021
Seeking instrumental support	−0.046	−0.052
Seeking emotional support	−0.065	−0.041
A turn to religion	0.016	0.109
Positive re-evaluation and development	−0.025	0.011
Refraining from action	0.302 ***	0.276 ***
Acceptance	−0.259 ***	0.226 ***
Focusing on emotions and their discharge	0.284 ***	0.194 **
Denial	0.330 ***	0.301 ***
Distraction	0.262 ***	0.236 ***
Cessation of activities	0.366 ***	0.500 ***
Use of alcohol or other psychoactive substances	0.198 ***	0.222 ***
Sense of humor	0.058	−0.165 **

*** *p* < 0.01; ** *p* < 0.05.

**Table 12:** Correlations between health problems and MiniCOPE scales.

	Chronic Illness	Depression/ Mood Disorders	Anxiety Disorders	Other Mental Illnesses
Active coping	0.061	−0.035	0.048	0.014
Planning	0.059	−0.045	0.050	0.021
Seeking instrumental support	0.196 ***	0.040	0.075	0.058
Seeking emotional support	0.105	−0.054	0.045	0.042
A turn to religion	0.06	−0.020	0.054	−0.059
Positive re-evaluation and development	0.072	−0.110	−0.065	0.037
Refraining from action	0.065	0.176 ***	0.200 ***	0.019
Acceptance	0.021	0.038	0.042	0.068
Focusing on emotions and their discharge	0.033	0.048	0.086	0.044
Denial	−0.032	−0.040	−0.037	0.074
Distraction	0.080	0.067	0.052	0.019
Cessation of activities	0.087	0.188 ***	0.136 **	−0.008
Use of alcohol or other means	−0.001	0.128 **	0.186 ***	0.158 ***
Sense of humor	0.062	−0.049	−0.044	0.076

*** *p* < 0.01; ** *p* < 0.05.

**Table 13:** Comparisons of the symptoms of anxiety and depression in participants with and without a previous COVID-19 diagnosis.

	COVID-19 Diagnosis		No COVID-19 Diagnosis				
	M	SD	M	SD	t	df	d
Anxiety	7.17	3.89	7.58	3.91	−0.85	279	0.11
Depression	4.95	3.73	5.40	3.71	−0.97	279	0.12

M—mean, SD—standard deviation, t—*t*-test value, df—degrees of freedom, d—Cohen’s effect size.

**Table 14:** Comparisons between symptoms of anxiety and depression in respondents working and not working in a COVID-19 ward.

	Yes/Yes, But No Longer Working		No		Not Applicable		
	M	SD	M	SD	M	SD	F
Anxiety	6.82	3.53	7.49	3.94	8.08	4.16	1.56
Depression	4.70	3.40	5.17	3.69	6.17	4.07	2.39

F—Fisher test for univariate ANOVA, M—mean, SD—standard deviation.

## Data Availability

The data presented in this study are available on request from the corresponding author.
